# MiR-1180 from bone marrow MSCs promotes cell proliferation and glycolysis in ovarian cancer cells via SFRP1/Wnt pathway

**DOI:** 10.1186/s12935-019-0751-z

**Published:** 2019-03-20

**Authors:** Jinghui Hu, Wei Zhao, Yujie Huang, Zhe Wang, Tingting Jiang, Li Wang

**Affiliations:** 1 Department of Gynaecology, The First Affiliated Hospital, School of Medicine, Zhejiang University, 79 Qingchun Road, Hangzhou, 310003 China; 2 Department of Gynaecology and Obstetrics, Changzhou Maternal and Child Health Care Hospital Affiliated Nanjing Medical University, Changzhou, China

**Keywords:** miR-1180, Ovarian cancer, Cell proliferation, Glycolysis, SFRP1

## Abstract

**Background:**

The ovarian cancer microenvironment is responsible for cancer cell growth and disease relapse. Bone marrow mesenchymal stem cells (BM-MSCs) play important roles in ovarian cancer, however, the mechanism of BM-MSCs inducing cell proliferation and glycolysis needs further research.

**Methods:**

miRNA array was used to analyze the significant miRNAs. RT-qPCR was used to examine the level of miR-1180 and SFRP1. The western blotting was used to detect the protein level of SFRP1 and Wnt signal pathway. We utilized luciferase reporter assay to confirm the direct interaction of SFRP1 with miR-1180. MTT assay were employed to investigate the proliferation of ovarian cancer cells. ECAR, ATP assay were used to measure the glycolysis state of ovarian cancer cells.

**Results:**

It was demonstrated that BM-MSCs promoted ovarian cancer cell proliferation and glycolysis. The miRNA profile from the BM-MSCs indicated that miR-1180 was up-regulated in the conditioned medium of BM-MSCs. MiR-1180 could accelerate ovarian cancer cell proliferation and glycolysis. We also found that up-regulation of miR-1180 activated Wnt signaling by targeting SFRP1 in ovarian cancer cells.

**Conclusion:**

The study demonstrated that miR-1180 was a critical miRNA mediating BM-MSCs induced cell proliferation and glycolysis and could be a new target in ovarian cancer therapy.

## Background

Mesenchymal stem cells are adult, self-renewing multipotent progenitors that construct the stromal compartment [1, 2]. Mesenchymal stromal/stem cell population (MSCs) is a population of stromal cells that demonstrate stem cell capabilities isolated from the bone marrow and from other diverse human tissues (like adipose, cartilage, muscle) [3–7]. Bone marrow mesenchymal stem cells (BM-MSCs) support tumor progression through immune suppression, epithelial-to-mesenchymal transition, angiogenesis, and serving as cancer stromal cells [8–12]. In contrast, BM-MSCs also suppress cancer by downregulating cancer survival signaling pathways involving WNT/β-catenin and/or AKT [8]. Ovarian cancer is the most common cancer from women, however, the effects of BM-MSCs on ovarian cancer are still unclear. It is to necessary to investigate the mechanisms underlying the contradictory roles of BM-MSCs on ovarian cancer cell biological functions.

In this study, we hypothesized that human BM-MSCs might have important influence on the regulation of ovarian cancer cell proliferation and glycolysis. Hence, we investigated the influence of BM-MSCs from miR-1180 on ovarian cancer cell glycolysis and cell proliferation. Our results showed that BM-MSCs treatment promoted cell glycolysis and cell proliferation of ovarian cancer cells. We also found that up-regulation of miR-1180 decreased SFRP1 expression, which activated Wnt signaling in ovarian cancer cells. Our results suggest that miR-1180 may be a therapeutic target in ovarian cancer.

## Methods

### Cell culture

All the ovarian cancer cell lines used in the study were primarily obtained from American Type Culture Collection (ATCC, Manassas, VA, USA). The cells were cultured according to the standard protocols. IOSE80 (normal ovarian epithelial cell line) cells were cultured in DMEM-F12 with 10% fetal bovine serum with penicillin (100 U/ml), streptomycin sulfate (100 µg/ml), EGF and insulin. The cells were incubated in a humidified incubator at 37 °C with 5% CO_2_.

### BM-MSCs isolation

BM was harvested from the sternum or iliac crest of seven healthy volunteers. Bone marrow was flushed out with 1 ml DMEM/F12 medium. The bone marrow was repeatedly washed to generate a single-cell suspension that was centrifuged at 1000 rpm for 5 min. The supernatant was removed, and cells were washed with DMEM/F12 and centrifuged for an additional 5 min. Finally, the supernatant was removed, and cells were resuspended in DMEM/F12 medium containing 10% fetal bovine serum (FBS) and 1% penicillin–streptomycin. Cells isolated from one hind limb were plated in a 25-cm^2^ dish and incubated at 37  °C with 5% CO_2_, which was defined as passage 0 (P0). After 24 h, cells were washed with PBS twice to remove non-adherent cells. When cell confluency was greater than 90%, the cells were secondarily cultured, and the passage number was increased by one.

### Conditioned medium preparation

Normal BM-MSCs (control) or the BM-MSCs co-cultured with ovarian cancer (BM-MSCs) were cultured in DMEM/F12 media with 10% FBS for 24 h, and then washed for three times with PBS and finally cultured in 3 ml serum free DMEM/F12 media for 2 h. Conditioned medium was collected and filtered through a 0.22-μm filter (Merck Millipore, Massachusetts, USA) to remove cellular debris for treating ovarian cancer cells.

### RNA isolation and miRNA array

The conditioned medium from Normal BM-MSCs (control) or the BM-MSCs co-cultured with ovarian cancer (BM-MSCs) was collected for total RNA extraction using TRIzol (Roche Applied Science). A three-step procedure was performed to profile the miRNAs. First, for cDNA synthesis from the miRNAs, 30 ng of total RNA was subjected to RT (reverse transcription) using a TaqMan^®^ microRNA Reverse Transcription Kit (Applied Biosystems) and Megaplex RT primers (Applied Biosystems) following the manufacturer’s protocol. RT was performed on a Mastercycler Epgradient thermocycler (Eppendorf) with the following cycling conditions: 40 cycles at 16 °C for 2 min, 42 °C for 1 min and 50 °C for 1 s followed by a final step of 80 °C for 5 min to inactivate reverse transcriptase. The expression profile of miRNAs was determined using the TaqMan^®^ Universal Master Mix II (Life Technologies, Applied Biosystems) in an Applied Biosystems 7900HT thermal cycler using the manufacturer’s recommended program. Finally, all the raw data from each array were retrieved from the 7900HT and run on Data Assist Software ver.3.1 (Applied Biosystems).

### Cell proliferation

Ovarian cancer cells were seeded in 6-well plates and transfected with miRNAs or treated with BM-MSCs conditioned medium and cultured in the normal condition. Cell survival ability was tested by the method of MTT assay.

### Colony formation assay

Ovarian cancer cells were seeded in 6-well plates. Cells were transfected miR-1180 in the present of BM-MSCs-CM and cultured in the normal condition. The cells were cultured for 10 days, washed with 1 × PBS, fixed with 70% ethanol for 5 min and stained with 0.5% crystal violet for 3 min at room temperature. The colonies (> 50 cells) were counted. All experiments were performed at least three times.

### ECAR

ECAR was measured in purified ovarian cancer cells following a 6-day culture in the presence or absence of stromal contact under basal conditions, in response to glucose, and upon blocking the mitochondrial ATP generation by oligomycin. The resulting (compensatory) effects on ECAR following the interference with the mitochondrial energy metabolism represent the maximal glycolytic capacity and are shown as a percentage of the baseline measurement (set as 100%).

### ATP levels

Adenosine triphosphate (ATP) concentration was assessed using a colorimetric ATP Assay Kit (Abcam, Cambridge, UK).

### RNA extraction and real-time PCR analysis

Ovarian cancer cells were transfected with miR-1180, anti-miR-1180 or the controls for 48 h and then total RNA was isolated for Real time RT-PCR analysis. The expression level of miRNAs was defined based on the threshold cycle (Ct), and relative expression levels were calculated using the 2^−ΔΔCt^ method, using the expression level of the U6 snRNA as a reference gene.

### Western blotting

Cultured cells were harvested and lysed with RIPA buffer containing the protease inhibitors on ice for 30 min. Equal protein was separated by SDS-PAGE. The protein was transferred onto nitrocellulose membrane using and probed with primary antibodies and then horseradish peroxidase-labeled secondary antibodies. The protein band signals were visualized using an ECL.

### Statistical analysis

The data were analyzed using the SPSS 18.0 (SPSS, Chicago, IL, USA) or Excel. Every experiment was completed independently at least three times. A p value < 0.05 was considered significant.

## Results

### BM-MSCs promotes ovarian cancer cell proliferation

**Fig. 1:**
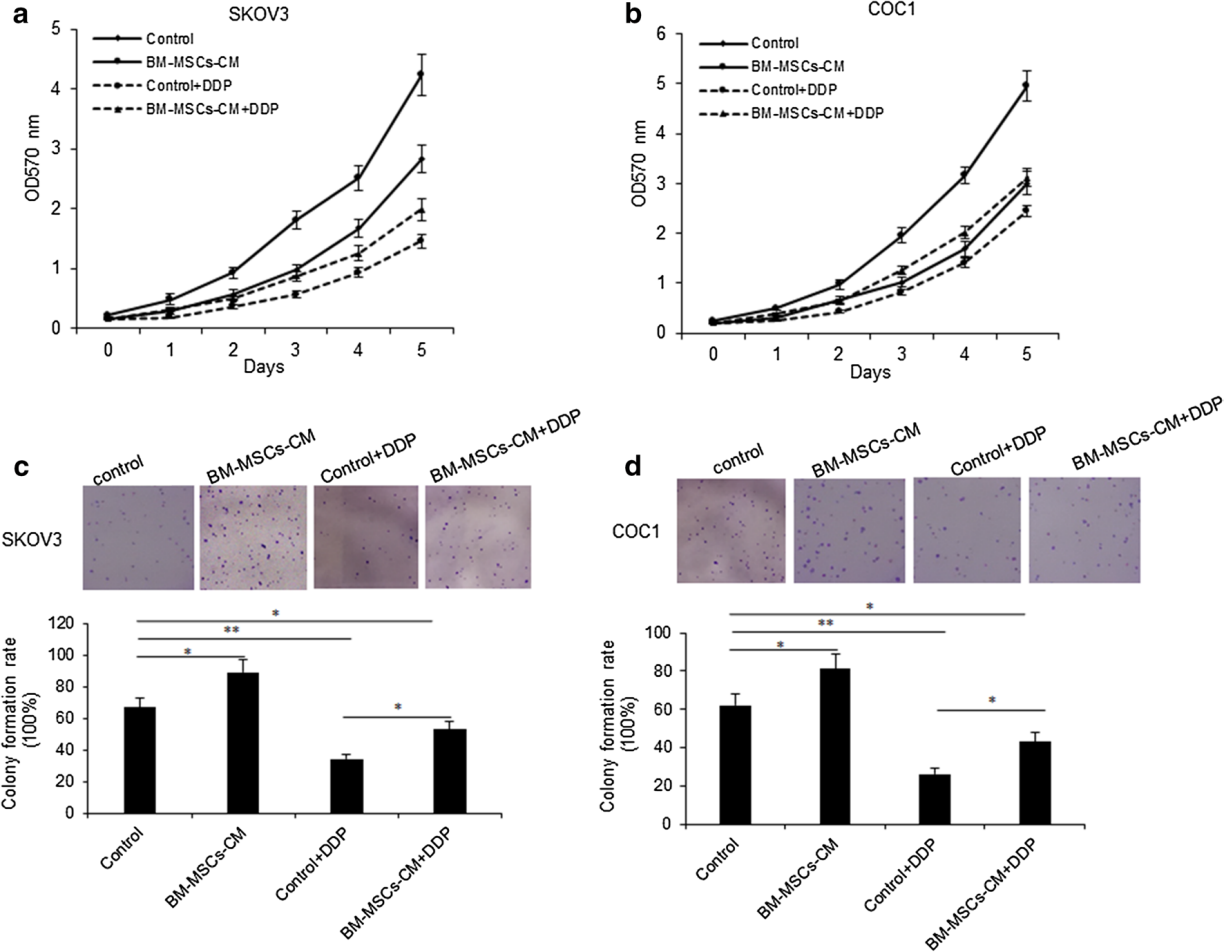
BM-MSCs promotes ovarian cancer cell proliferation. **a**, **b** SKOV3 and COC1 ovarian cancer cell proliferation was assayed by MTT method. SKOV3 and COC1 cells were exposed to BM-MSCs-CM for 24 h. Cell survival rates were analyzed at 1, 2, 3, 4 and 5 days. **c**, **d** SKOV3 and COC1 ovarian cancer cell survival ability was assayed by colony formation assay. SKOV3 and COC1 cells were treated with BM-MSCs-CM for 24 h, and then seeded in 6 cm culturing plates. The cells were cultured for 10 days and the colonies were counted. The up panels showed the photos of the colony and the low panels showed the analyzed data of the colony numbers from the photos. **p < 0.01; *p < 0.05

To investigate the role of BM-MSCs in ovarian cancer cell functions, conditioned medium (CM) was collected from serum-free medium of BM-MSCs through a centrifugation and a filtration step. And then SKOV3 and COC1 cells were treated with BM-MSCs derived CM with or without cisplatin (DDP) combination and cell proliferation was assayed by CCK8. The data showed that BM-MSCs-CM promoted cell proliferation of SKOV3 and COC1 cells (Fig. 1a, b). We used colony formation assay to verify the result (Fig. 1c, d). These data demonstrated that BM-MSCs-CM promoted ovarian cancer cell growth.

### BM-MSCs promoted ovarian cancer cell glycolysis

**Fig. 2:**
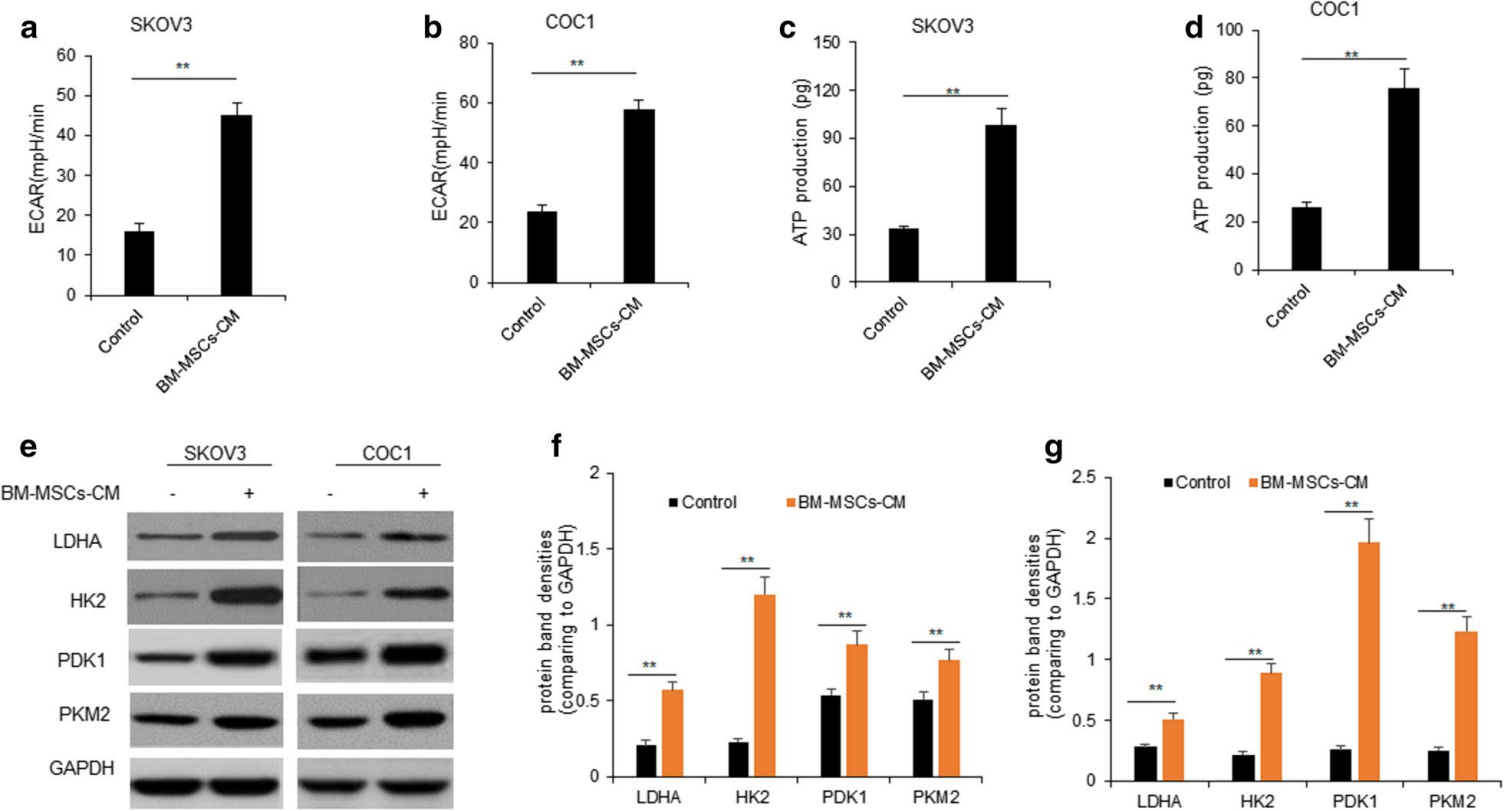
BM-MSCs promoted ovarian cancer cell proliferation and glycolysis. **a**, **b** SKOV3 and COC1 ovarian cancer cell ECAR was assayed. SKOV3 and COC1 cells were in the present of BM-MSCs-CM for 1 days and then for ECAR analysis. **c**, **d** ATP production in SKOV3 and COC1 ovarian cancer cell was assayed. SKOV3 and COC1 cells were in the present of BM-MSCs-CM for ATP production. **e** Glycolysis protein was examined in SKOV3 and COC1 ovarian cancer cells. SKOV3 and COC1 cells were treated with in the present of BM-MSCs-CM for 1 days and then the cells were collected for glycolysis associated protein analysis by western blotting. **f**, **g** The bands from **e** were quantified. **p < 0.01; *p < 0.05

In order to evaluate the impact of BM-MSCs on ovarian cancer cell glycolysis, we measured ECAR of ovarian cancer cells with BM-MSCs-CM treatment. Ovarian cancer cells treated with BM-MSCs-CM led to a marked increase the extracellular acidification rate (ECAR) of SKOV3 and COC1 cells (Fig. 2a, b). ATP production was also detected in SKOV3 and COC1 cells. The results showed that ATP production in cells increased with BM-MSCs-CM treatment (Fig. 2c, d). Consequently, the expression levels of key enzymes controlling glycolysis including LDHA, HK2 and PKM2 were significantly higher in SKOV3 and COC1 cells with BM-MSCs-CM treatment than the controls (Fig. 2e–g). So, BM-MSCs played a role in the ovarian cancer cell glycolysis.

### MiR-1180 was up-regulated in the conditioned medium of BM-MSCs

**Fig. 3:**
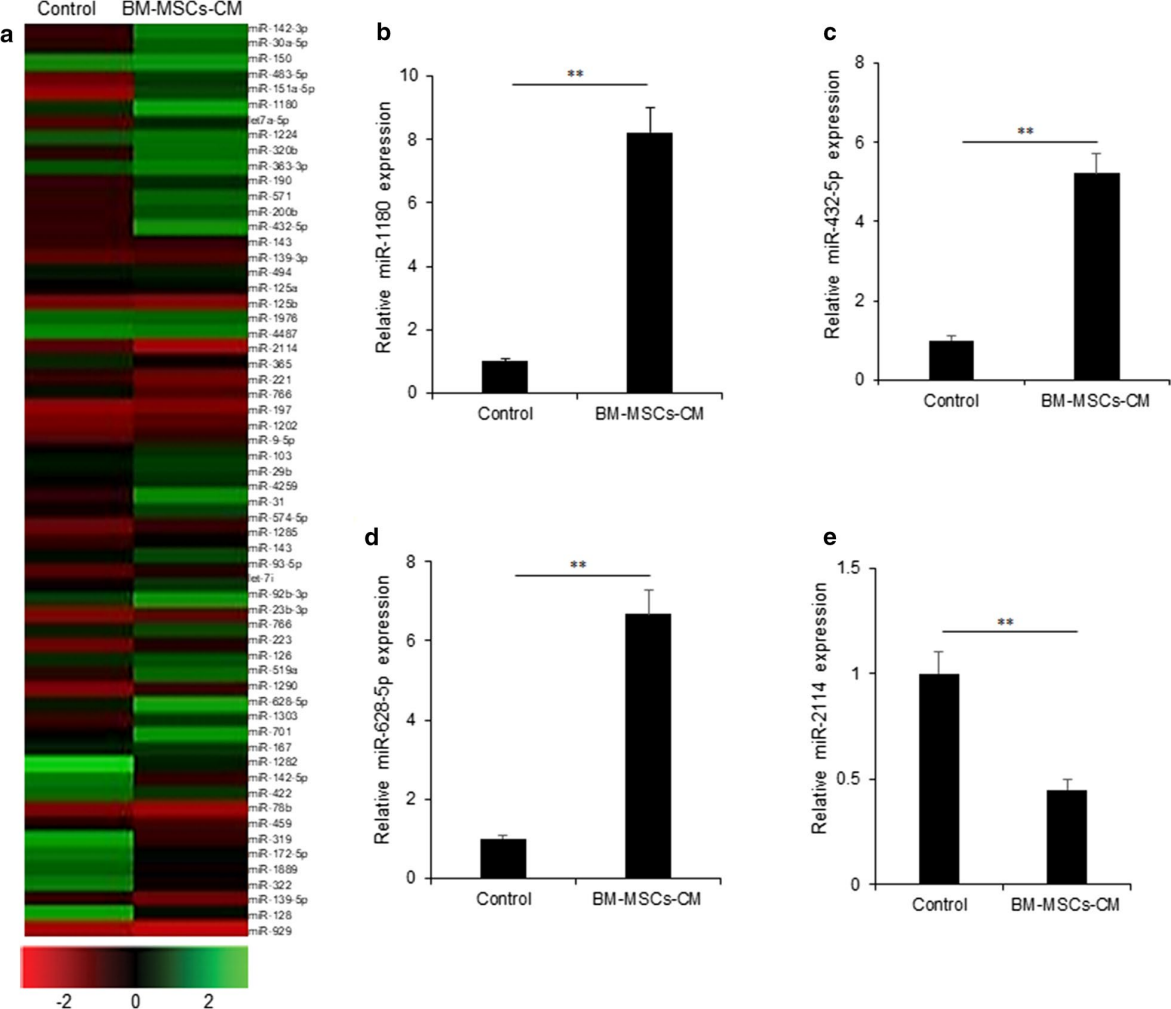
MiR-1180 was up-regulated in the conditioned medium of BM-MSCs. **a** MiRNA expression map. **b** miR-1180 expression were selected for real time RT-PCR verification in BM-MSCs. **c** miR-628-5p expression were selected for real time RT-PCR verification in BM-MSCs. **d** miR-432-5p expression were selected for real time RT-PCR verification in BM-MSCs. **e** miR-2114 expression were selected for real time RT-PCR verification in BM-MSCs. **p < 0.01

To further explore the mechanism of BM-MSCs promoting ovarian cancer cell proliferation and glycolysis, the conditioned medium from BM-MSCs were performed for miRNA array analysis. The miRNA array was shown in Fig. 3a. The miRNA profile showed that there were up-regulated miRNAs in BM-MSCs like miR-1180, miR-628-5p, miR-432-5p and down-regulated miRNAs like miR-2114, miR-78b and etc. Some of significant miRNAs from the array were selected for real time RT-PCR analysis and it was found that miR-1180 was significantly up-regulated in CM from BM-MSCs (Fig. 3b). MiR-628-5p and miR-432-5p were confirmed to be up-regulated (Fig. 3c, d) and miR-2114 was down-regulated (Fig. 3e) in BM-MSCs-CM.

### MiR-1180 contributed to ovarian cancer cell proliferation and glycolysis

**Fig. 4:**
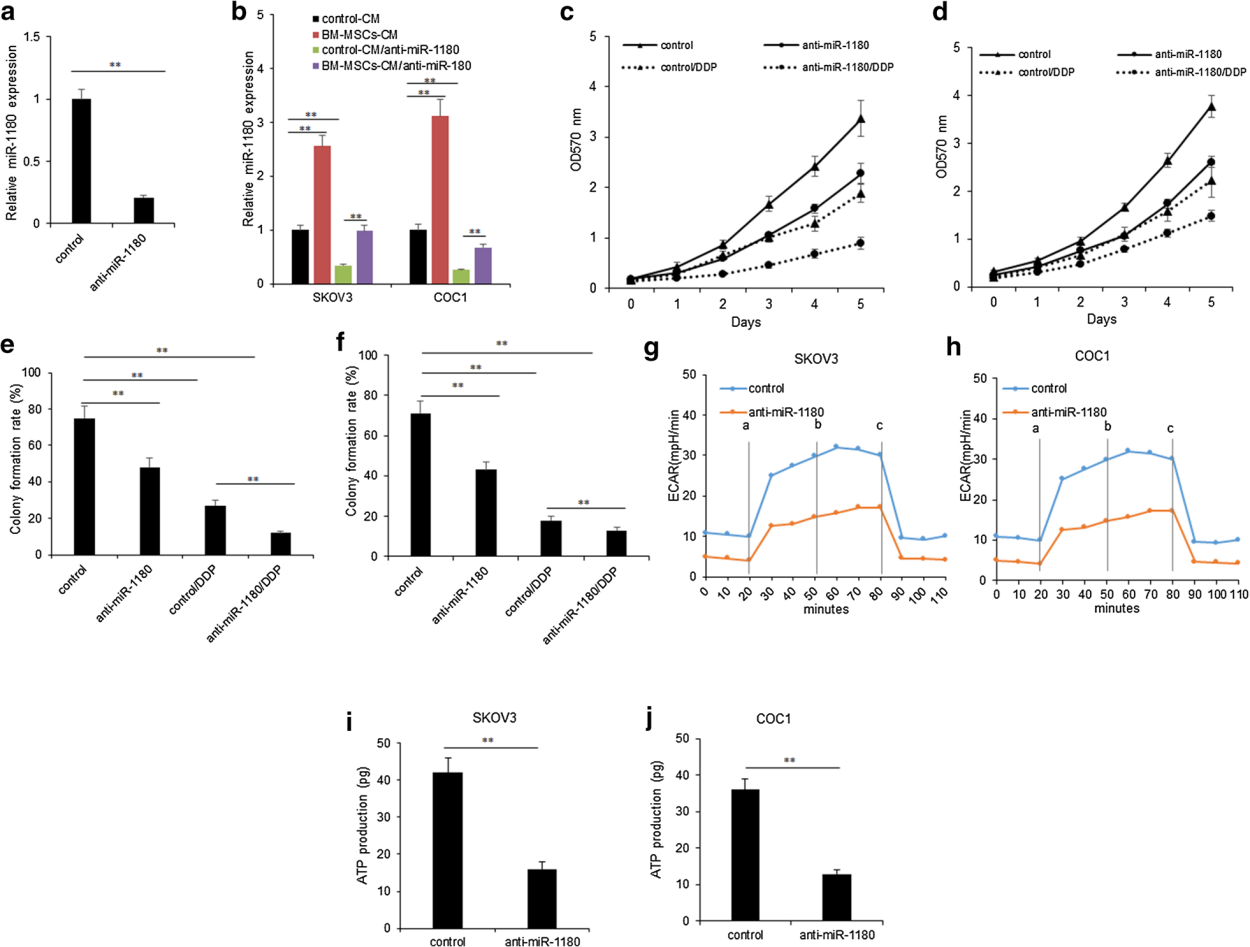
MiR-1180 contributed to ovarian cancer cell proliferation and glycolysis. **a** miR-1180 expression in BM-MSCs. BM-MSCs were transfected with miR-1180 inhibitors or the miRNA inhibitor controls for 48 h and the RNA was extracted for real time RT-PCR. **b**–**d** SKOV3 and COC1 cell proliferation was assayed by MTT method. SKOV3 and COC1 cells were treated with the conditioned medium (CM) from the BM-MSCs with miR-1180 down-regulation by the inhibitors transfection. Cell growth was measured at day 1, 2, 3, 4 and 5. **e**, **f** SKOV3 and COC1 cell survival ability was assayed by colony formation assay. SKOV3 and COC1 cells were treated with the conditioned medium (CM) from the BM-MSCs with miR-1180 down-regulation by the inhibitors transfection. The cells were cultured for 10 days and the colonies were counted. **g**, **h** SKOV3 and COC1 ovarian cancer cell ECAR was assayed. SKOV3 and COC1 cells were treated with the conditioned medium (CM) from the BM-MSCs with miR-1180 down-regulation by the inhibitors transfection for ECAR analysis. a: glucose; b: Oligomycin; c: 2-DG. **i**, **j** ATP production in SKOV3 and COC1 ovarian cancer cell was assayed. SKOV3 and COC1 cells were treated with the conditioned medium (CM) from the BM-MSCs with miR-1180 down-regulation by the inhibitors transfection for ATP production analysis. **p < 0.01; *p < 0.05

In order to evaluate the impact of miR-1180 on the cellular survival ability, drug response and glycolysis of ovarian cancer cells, firstly, miR-1180 expression was knocked down in BM-MSCs. It was found that miR-1180 expression was down-regulated in BM-MSCs (Fig. 4a). When SKOV3 and COC1 cells were treated with the CM from BM-MSCs with miR-1180 down-regulation, miR-1180 was also decreased (Fig. 4b). Next, the cell proliferation was assayed by MTT. SKOV3 and COC1 cells were treated with the CM from BM-MSCs with miR-1180 down-regulation or the control in the present DDP, and it was shown that BM-MSCs with miR-1180 down-regulation inhibited cell proliferation and also enhanced the DDP sensitivity (Fig. 4c, d). Cell colony formation assay showed that BM-MSCs with miR-1180 down-regulation reduced the colonies and also enhanced the DDP sensitivity (Fig. 4e, f). To evaluate the impact of miR-1180 on ovarian cancer cell glycolysis, we measured ECAR of ovarian cancer cells treated with the CM from BM-MSCs with miR-1180 down-regulation. BM-MSCs with down-regulation of miR-1180 led to a marked decrease the ECAR in SKOV3 and COC1 cells (Fig. 4f, g). There were shown that ATP production was suppressed in SKOV3 and COC1 cells with in SKOV3 and COC1 cells (Fig. 4h, i).

### MiR-1180 activated Wnt signal pathway in ovarian cancer cells by targeting SFRP1

**Fig. 5:**
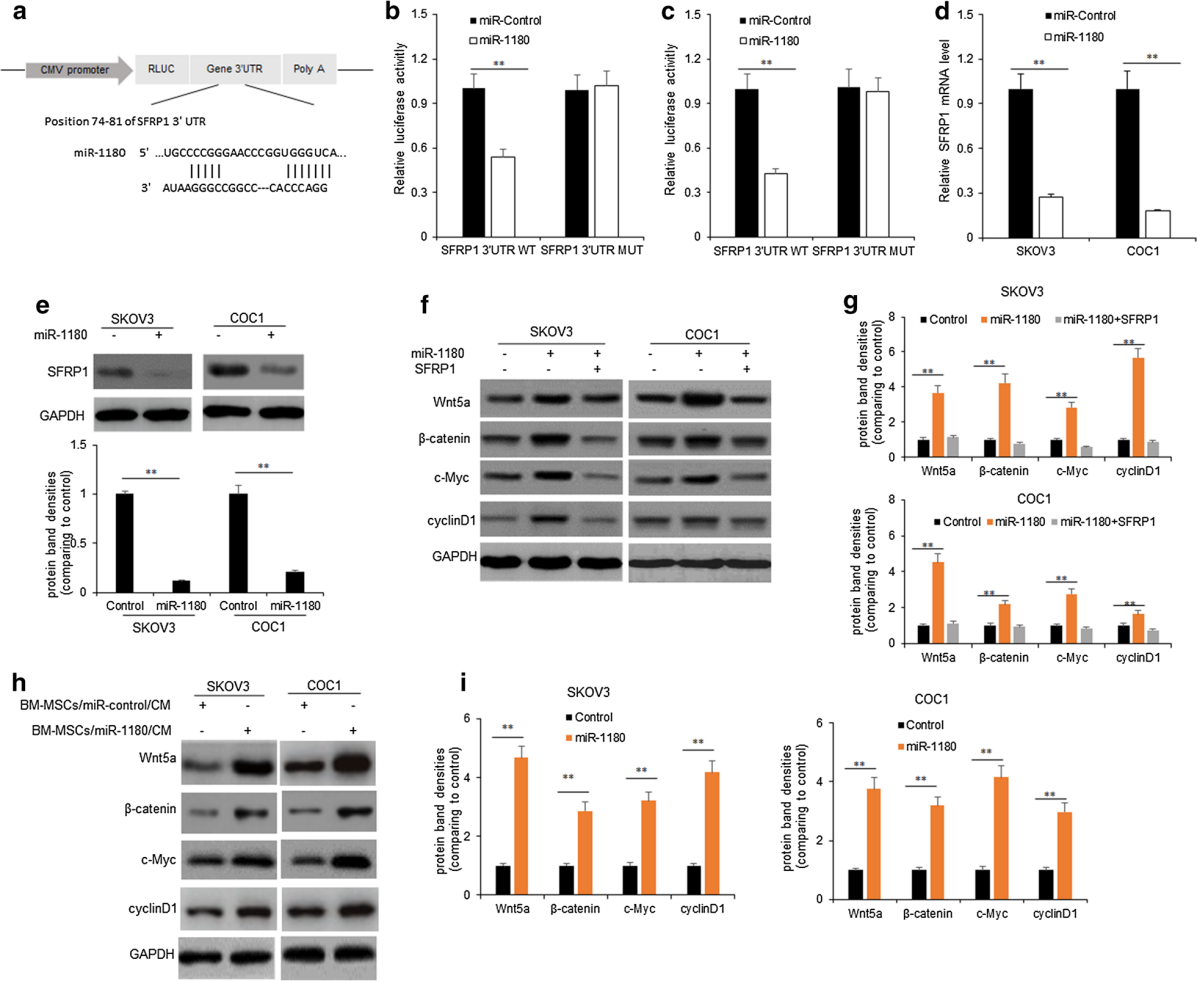
MiR-1180 activates Wnt signal pathway in ovarian cancer cells by targeting SFRP1. **a** Schematic representation of 3′-UTR of mRNA reporter with the miR-1180 seed-binding sites. **b**, **c** Luciferase activity assay of SKOV3 and COC1 cells transfected with luciferase constructs containing WT-3′UTR and Mut-3′UTR of SFRP1. **d** SFRP2 mRNA was determined by qPCR in SKOV3 and COC1 cells transfected with miR-1180 mimics or their controls. **e** SFRP1 protein was determined by western blotting in SKOV3 and COC1 cells transfected with miR-1180 mimics or their controls. **f** Wnt signal pathway associated protein was determined by western blotting in SKOV3 and COC1 cells transfected with miR-1180 mimics or SFRP1. **g** Protein band density from F. The protein band densities from Wnt5a, β-catenin, c-Myc and cyclinD1 were calculated. **h** Wnt signal pathway associated protein was determined by western blotting in SKOV3 and COC1 cells treated with the condition medium (CM) from BM-MSCs with miR-1180 mimics transfection. **i** Protein band density from H. The protein band densities from Wnt5a, β-catenin, c-Myc and cyclinD1 were calculated. **p < 0.01; *p < 0.05

To find the target genes of miR-1180 which are associated with cell proliferation and glucose metabolism, miRNA target gene prediction tools like Targetscan were used to predict the target genes of miR-1180. We found that SFRP1 was a target gene of miR-1180 (Fig. 5a). Next, wild-type 3′UTR SFRP1 (WT-3′UTR) or mutant 3′UTR SFRP1 (Mut-3′UTR) were constructed using the reporter vector carrying luciferase. To know whether miR-1180 directly binds the 3′UTR region of SFRP1, miR-1180 mimics and the SFRP1 WT-3′UTR or Mut-3′UTR, the result showed that the luciferase activity of SFRP1WT-3′UTR was inhibited by miR-1180 in SKOV3 and COC1 cells respectively (Fig. 5b, c). To further confirm whether miR-1180 regulate SFRP1 expression on post-transcriptional levels, SKOV3 and COC1 cells were transfected with miR-1180 mimics and miRNA controls, SFRP1 mRNA and protein were evaluated by real time RT-PCR and western blotting respectively. The data showed that SFRP1 mRNA was down-regulated in SKOV3 and COC1 cells (Fig. 5d). The protein levels of SFRP1 from the cells with miR-1180 transfection decreased (Fig. 5e).

SFRP1 is a negative regulator of Wnt signal pathway. To explore the possible relationship between miR-1180 and Wnt signal pathway ovarian cancer cells, SKOV3 and COC1 cells were treated with miR-1180 mimics and SFRP1 for Wnt signal pathway associated protein immunoblotting. The result indicated that miR-1180 could activate Wnt signal pathways because Wnt5a, β-catenin, myc and cyclinD1 levels increased in ovarian cancer cells with miR-1180 and SFRP1 overexpression (Fig. 5f, g). When SKOV3 and COC1 cells were treated with the conditioned medium from BM-MSCs with miR-1180 expression, Wnt5a, β-catenin, myc and cyclinD1 protein levels increased comparing with the cells treated with the conditioned medium from BM-MSCs without miR-1180 expression (Fig. 5h, i).

### MiR-1180 was a potential diagnostic marker and negatively related to SFRP1 expression in ovarian cancer

**Fig. 6:**
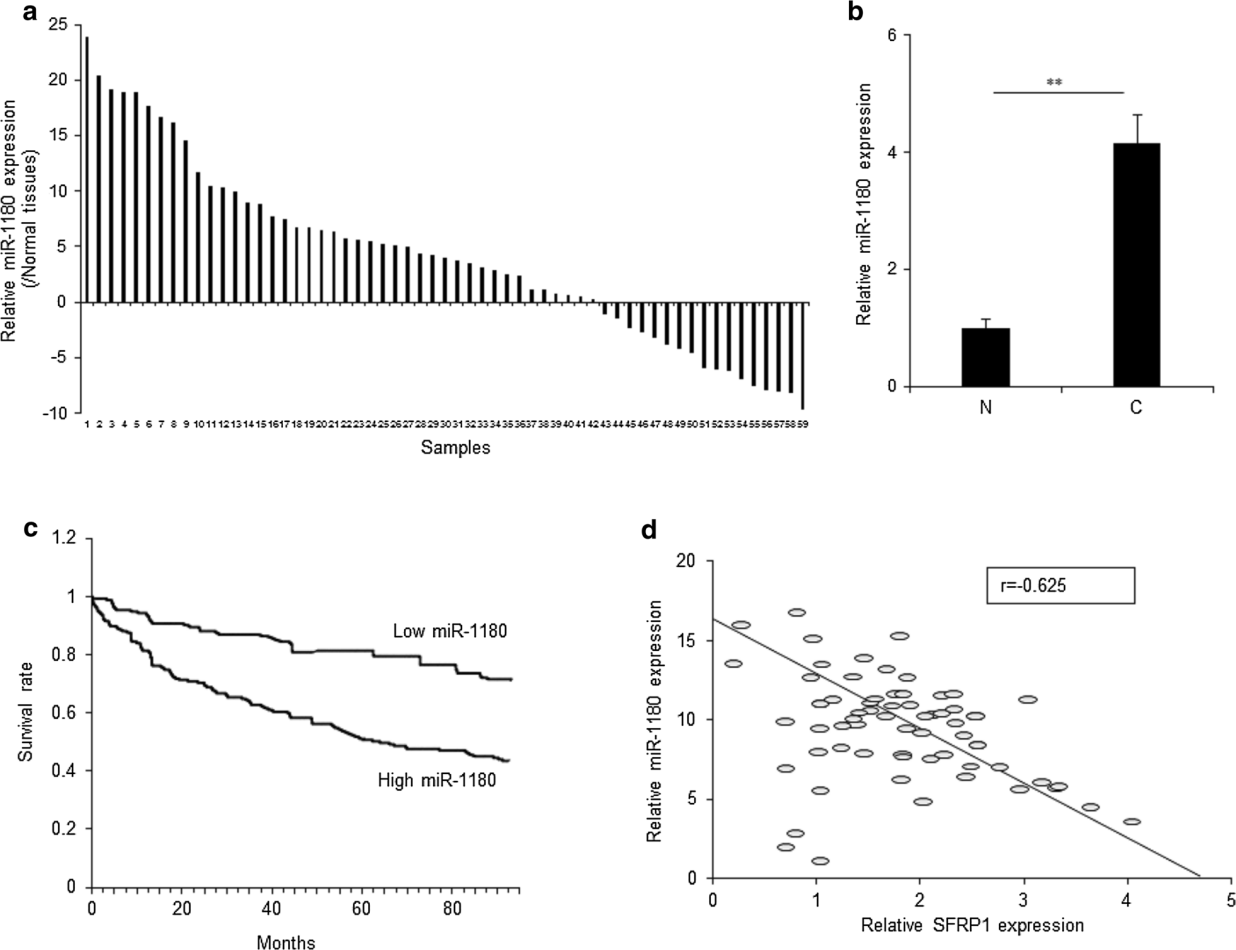
MiR-1180 was a potential the diagnostic marker and negatively related to SFRP1 expression in ovarian cancer. **a** miR-1180 expression in ovarian cancer tissues (n = 59) and adjacent normal tissues (n = 59). 59 ovarian cancer tissues were used for miR-1180 expression detection by qRT-PCR. **b** The average miR-1180 expression from ovarian cancer tissues and adjacent normal tissues. Data from A was analyzed. **c** The survival rate of ovarian cancer with higher miR-1180 levels (n = 34) and lower miR-1180 levels (n = 25). **d** The miR-1180 expression was negatively related to SFRP1 expression in ovarian cancer tissues. **p < 0.01

To explore whether miR-1180 is the diagnostic marker of ovarian cancer, miR-1180 levels were examined in the tissues of ovarian cancer. The result showed that miR-1180 levels were higher in ovarian cancer tissues than the adjacent normal tissues (Fig. 6a). The average of miR-1180 levels in ovarian cancer tissues was higher than the adjacent normal tissues (Fig. 6b). The survival rate of ovarian cancer with higher miR-1180 expression was shorter than the patients with lower miR-1180 levels (Fig. 6c). The relationship between miR-1180 and SFRP1 showed that miR-1180 expression was negatively related to SFRP1 mRNA levels in ovarian cancer tissues (Fig. 6d).

## Discussion

MSCs in tumor microenvironment are from bone marrow or other tissues. MSCs integrate into the tumor stroma and function in a paracrine manner to promote ovarian cancer progression. The secreting paracrine molecule is a main functional way in tumor stroma [5–8]. Although there are studies indicating the important roles of BM-MSCs in ovarian cancer, the roles of MSCs in cell proliferation and glycolysis is still unclear. The molecular mechanisms mediating ovarian cancer cell glycolysis need to be investigated. In this study, our purpose is to investigate the effects of BM-MSCs on ovarian cancer cell glycolysis. We found that MSCs could promote ovarian cell survival ability and glycolysis via the up-regulation of miR-1180/Wnt signaling pathway.

The reported studies show that MSCs exert a positive effect on cancer cell growth [1, 2, 13, 14]. Unlimited cell proliferation is a most characteristics of malignant tumor. So, BM-MSCs were isolated and co-cultured with ovarian cancer cell to observe cell proliferation. It was found that BM-MSCs promoted ovarian cancer cell proliferation. The Warburg effect is a characteristic of cancer. We measured ECAR and ATP production of ovarian cancer cells with BM-MSCs conditioned medium. The data clearly demonstrated that MSCs increased ovarian cancer cell glycolysis.

MiRNAs play great important roles in ovarian cancer glycolysis. MiR-1180 is reported as a tumor suppressive miRNA in bladder cancer cells and inhibits cell proliferation and tumorigenicity by inhibiting cell cycle related proteins including CDK4, CDK6, cyclinD1, cyclinA2 expression and up-regulating p21 expression [15]. However, another report showed that miR-1180 is up-regulated in hepatocellular carcinoma [16, 17]. MiR-1180 promotes hepatocellular carcinoma cell proliferation by down-regulating TNIP2 expression [16] and induces apoptosis-resistance by activating NF-κB signaling pathway [17]. So, miR-1180 is a tumor promoter or a tumor suppressor depending on the cancer phenotype and genetics background. Our study showed that miR-1180 expression in BM-MSCs conditioned medium was up-regulated. Further study identified that SFRP1 was a direct target gene of miR-1180 in MSCs. We found that miR-1180 promoted glycolysis via targeting SFRP1. SFRP1 is reported as a tumor suppressor in breast cancer [18, 19]. Wnt signal pathway is activated in cancers by loss of SFRP1 expression [20, 21]. In addition, Wnt signal pathway is related to cancer cell drug resistance [22–26] and glycolysis [27–30]. Our findings indicated that BM-MSCs promoted ovarian cancer cell proliferation and glycolysis by miR-1180 activating Wnt pathway.

In the present study, our findings indicated that BM-MSCs promoted ovarian cancer cell proliferation and glycolysis by miR-1180. MiR-1180 functioned as onco-miRNA by activating Wnt pathway. There needs further research on the mechanisms of MSCs derived miR-1180 in ovarian cancer progression. Our study for the first time verified that miR-1180 functioned as an onco-miRNA in ovarian cancer cell by targeting SFRP1 expression in ovarian cancer cells.

## Conclusion

The data from our study showed that BM-MSCs treatment promoted cell glycolysis and cell proliferation of ovarian cancer cells. We also found that up-regulation of miR-1180 decreased SFRP1 expression, which activated Wnt signaling in ovarian cancer cells. Our results suggest that miR-1180 may be a therapeutic target in ovarian cancer.

## Abbreviations

RT-qPCR: reverse transcription quantitative real time polymerase chain reaction
3′-UTR: 3′-untranslated region
BM-MSCs: bone marrow mesenchymal stem cells
ATCC: American Type Culture Collection
MTT: 3-(4,5-dimethyl-2-thiazolyl)-2,5-diphenyl-2-*H*-tetrazolium bromide
RIPA: radio immunoprecipitation assay
SDS-PAGE: sodium dodecyl sulfate-polyacrylamide gel electrophoresis
ECL: enhanced chemiluminescence
ATP: adenosine triphosphate
ECAR: extracellular acidification rate
